# Bullying roles, moral disengagement, and motivational perceptions among university students

**DOI:** 10.3389/fsoc.2024.1511340

**Published:** 2025-01-16

**Authors:** Sohni Siddiqui, Anja Schultze-Krumbholz, Muhammad Kamran

**Affiliations:** ^1^Department of Educational Psychology, Technical University of Berlin, Berlin, Germany; ^2^Department of Education, University of Loralai, Loralai, Pakistan

**Keywords:** bullying groups, moral disengagement beliefs, perceptions about motivations behind bullying, university students in Pakistan, peer and teacher interventions

## Abstract

**Introduction:**

Bullying is a significant social problem that affects educational institutions worldwide, including those in Pakistan. This study extends the existing literature by going beyond reporting the prevalence and consequences of bullying in Pakistan. It examines the prevalence of different bully groups among university students (*N* = 1,034; male = 361; female = 665) and explores the relationships between their characteristics, moral disengagement beliefs, and perceptions about motivations for bullying perpetration.

**Methods:**

The present study used a cross-sectional design. Descriptive analyses, Pearson correlations, one-way ANOVA, and independent t-tests were conducted.

**Results:**

Approximately one-quarter of students identified themselves as victims only, while 14% of students identified themselves as both victims and perpetrators of bullying. Most students reported frustration as the primary motive for engaging in aggressive acts. In addition, students with higher moral disengagement beliefs were more likely to engage in bullying behavior, with the highest correlation observed between moral disengagement and bullying as a means of demonstrating power and superiority. Significant differences in perceived motivations for bullying were also observed between the victim and bully-victim groups. Analyses showed that male students were more likely to be involved in bullying others, while female students showed higher levels of fear of victimization within educational institutions.

**Discussion:**

The results implicate the need for innovations in programs and the inclusion of moral disengagement measures. Identifying the key mechanisms underlying behavioral change away from bullying should be a central focus of anti-bullying prevention and intervention programs.

## 1 Introduction

Bullying is the intentional and repeated harassment of an individual through physical, psychological, or verbal means (Olweus, 2013). The adverse effects associated with bullying victimization include both physical problems such as headaches, sleep disturbances, stomachaches, depression and mental health problems such as anxiety disorders, psychological distress somatic disorders, and low psychological wellbeing (Eyuboglu et al., 2021; Fullchange and Furlong, 2016; Grinshteyn et al., 2021; Lin et al., 2020; Yosep et al., 2022). Bullying is often accompanied by derogatory language, and its typical goal is to demean the victim while trying to impress peers (Rose et al., 2017). This behavior is usually intended to cause harm or embarrassment to the victim. Bullying can manifest as physical, psychological, emotional, or cyber-based aggression perpetrated by students who use their power to harm more vulnerable peers (Helgeland and Lund, 2017; Siddiqui and Schultze-Krumbholz, 2023a). In addition, research has documented the negative impact of bullying on the academic trajectories of victimized students (Yosep et al., 2022). It is a significant social ill affecting educational institutions worldwide (Jones et al., 2023), including those in Pakistan (Batool, 2023; Razzaq et al., 2023; Srinivasan et al., 2022). There is an urgent need to develop interventions that focus on the root causes of the problem in order to mitigate it at its core (Siddiqui et al., 2023b), including the impact of moral disengagement beliefs and identifying the perceptions of the motivations behind bullying perpetration (Siddiqui and Schultze-Krumbholz, unpublished study)^1^.

The bullying literature has increasingly focused on examining bullying behaviors and has identified four distinct types: pure bullies, pure victims, bully-victims, and neither-bully-nor-victim/non-responsive (Johnston et al., 2014). Most research has focused on pure bullies and pure victims (Johnston et al., 2014). However, this study not only examines the prevalence of different involvement groups among university students in Pakistan, but also explores the relationship between their characteristics and moral disengagement beliefs. It also examines differences in their perceptions of the motivations behind bullying perpetration. The prevalence, types of bullying, and involvement in different bullying roles reported by researchers vary across age groups, as found in studies conducted worldwide (Gini et al., 2014; Menesini and Salmivalli, 2017; Modecki et al., 2014; Runions et al., 2019; Siddiqui et al., 2021; Siddiqui and Schultze-Krumbholz, 2023a; Thornberg and Jungert, 2014; UNESCO, 2019). In addition, international research highlights differences in motivations for bullying perpetration across age groups and regions (Abbasi et al., 2018; Fluck, 2017; Jaber et al., 2023; Johnston et al., 2014; Tam and Taki, 2007; Tanrikulu and Erdur-Baker, 2021; Sarıçam and Çetinkaya, 2018; Schreiner, 2019; Van Cleave and Davis, 2006; van Dijk et al., 2017; Xiu et al., 2021). A longitudinal study by Zych et al. (2020) found that the roles of victim and bully tend to be unstable over time, while the role of bully-victim changes more significantly with age. However, the relationship between age and bullying motivations is relatively underexplored and varies across cultures. Nevertheless, a recent study by Siddiqui and Schultze-Krumbholz (unpublished study) reported that university students often engage in bullying for the thrill and excitement it provides.

Numerous international studies have found that individuals with higher levels of moral disengagement are more likely to engage in aggressive behavior, but their motivations for antisocial behavior vary widely (Killer et al., 2019; Romera et al., 2021; Teng et al., 2020; Thornberg et al., 2019). However, there is a lack of research on this topic in the Pakistani context. Furthermore, international research has extensively explored the role of teachers and peers in mitigating bullying incidents within institutions, but these studies primarily focus on younger students (Siddiqui and Schultze-Krumbholz, 2023b; Zambuto et al., 2020; Zhao and Chang, 2019). To address this gap, the present study examines the relationship between moral disengagement and perceptions of bullying motives, as well as the role of teachers and peers in bullying intervention at the university level.

The primary objectives of this study were to identify the prevalence of different bullying roles among university students, to examine differences in perceptions of bullying motives by bullying roles, and to analyze the association of moral disengagement with perceptions of motives. In addition, other characteristics of bullying situations were also examined, such as the frequency of group bullying, the tendency to perpetuate, the role of teachers and peers in bullying incidents, and students’ fear of attending school. This study used descriptive statistics to explore the status of teacher and peer interventions in the university context in Pakistan. The final section of this study is dedicated to gender differences in these dynamics.

This study represents the first comprehensive attempt in Pakistan to explore the different roles that students play in bullying and the differences in their perceived motivations. As universities are also educational institutions, which moreover have so far often been neglected in bullying research, the authors chose to focus on university students to gain a deeper understanding of this particular age group, given their impending transition into professional roles. It has also been reported that students involved in bullying during their education often transition into adult bullying roles in the workplace (Reknes et al., 2021). It is therefore crucial to target this age group during this final stage of education, as it provides a crucial opportunity to guide potential perpetrators to avoid bullying behavior and to empower victims to stand up for themselves. In professional careers, opportunities for behavior change and improvement are severely limited. This study is of paramount importance because it not only assists higher education institutions in determining the prevalence of bullying, victimization, and bully-victim dynamics, but also elucidates the underlying motivations. This insight can assist faculty and counselors in developing more effective prevention strategies (Johnston et al., 2014; Siddiqui and Schultze-Krumbholz, unpublished study). This research is particularly novel in the Pakistani context, where bullying research is still in its infancy.

### 1.1 Definitions of key terms

- *Bullying:* Bullying is repeated, intentional behavior in which an individual or group attempts to harm, intimidate, or exert power over another person who is unable to defend himself or herself.
- *Bullying perpetration:* Bullying perpetration refers specifically to the act of engaging in bullying behavior, i.e., being the person or group responsible for initiating or carrying out bullying.
- *Bullying victimization:* Bullying victimization refers to the experience of being the target of bullying behavior by others.
- *Motives for bullying:* The motivations for bullying refer to the underlying reasons or drives that lead individuals to engage in bullying behavior.
- *Bullying roles:* Bullying roles refer to the specific positions or functions that individuals assume within the dynamics of a bullying situation. The four main bullying roles used and discussed in this study are the bully (perpetrator) only, the victim only, the bully/victim and the non-involved (bystander).

## 2 Literature review

### 2.1 Moral disengagement beliefs and bulling behaviors

The moral disengagement framework explains how individuals can engage in behaviors they know are morally wrong without experiencing remorse, guilt, or other self-sanctioning emotions (Bandura et al., 1996). It is conceptualized as a set of socio-cognitive mechanisms, or cognitive reframing strategies, that allow individuals to deactivate self-sanctions such as shame, guilt, and negative self-evaluation that would typically result from violating one’s own moral standards (Bandura et al., 1996). Several studies have confirmed that children and adults with higher levels of moral disengagement beliefs exhibit increased aggression and a greater tendency to engage in bullying behaviors (Killer et al., 2019; Teng et al., 2020; Thornberg et al., 2019). Similarly, Romera et al. (2021) found that children and adolescents with elevated moral disengagement beliefs were primarily motivated to engage in bullying behaviors to gain popularity. Numerous studies have been conducted to identify differences in moral disengagement beliefs among bullies, victims, and bully-victims (Gini et al., 2014; Runions et al., 2019; Thornberg and Jungert, 2014). However, there is a dearth of literature and research on this topic from Pakistan. Furthermore, the influence of moral disengagement on different perceived motivations for bullying has not been extensively explored in the Pakistani context. Therefore, this research was designed to address these gaps.

### 2.2 Prevalence of bullying and victimization in Pakistan

Bullying is a pervasive problem that occurs in many settings, including schools (Siddiqui and Schultze-Krumbholz, 2023a). According to the United Nations Educational, Scientific and Cultural Organization (UNESCO), one in three children worldwide is bullied. Specifically, 15.3 percent of children were bullied because of their appearance, 10.9 percent because of their race, color, or country of origin, and 4.6 percent because of their religion (UNESCO, 2019). Similarly, bullying in Pakistan is deeply entrenched and widespread in educational institutions, as shown by several studies (Ahmed et al., 2023; Mubasher et al., 2023; Murad, 2022; Perveen et al., 2022; Rauf et al., 2022; Siddiqui et al., 2021; Siddiqui and Schultze-Krumbholz, 2023a). Ayub et al. (2022) reported that approximately 70% of postgraduate trainees experienced bullying at their institution. Similarly, Saleem et al. (2021) found that approximately 90% of university students experienced cyberbullying. Continuing this trend, Siddiqui and Schultze-Krumbholz (2023a) found that approximately 98% of faculty had witnessed or reported incidents of bullying at their institutions. Gender differences in bullying perpetration and victimization have been documented in numerous international studies (Iyanda, 2022; Malecki et al., 2020; Prasartpornsirichoke et al., 2022), as well as within Pakistani society (Khawar and Malik, 2016), where women are more often targeted for bullying victimization (Magsi et al., 2017; Zheng et al., 2024). Magsi et al. (2017) found that women in universities experience ridicule and harassment through electronic platforms, and about half of the victims do not report these incidents due to cultural and religious constraints or to avoid being blamed. As a result, many women suffer in silence and may withdraw from online activities as a means of self-protection. Similarly, Zheng et al. (2024) reported that workplace bullying is a significant reason why female breadwinners choose to leave their jobs. In a study of children and adolescents by Rauf et al. (2022), males were more likely to be involved in bullying perpetration than females. Meanwhile, females tend to have more mental health problems than males. On the other hand, a study by Begum et al. (2019) found no significant gender differences in bullying behavior. Despite, the confirmed high prevalence of bullying in educational institutions in Pakistan, there remains a lack of literature on effective interventions to mitigate bullying and improve the educational environment (Siddiqui et al., 2023a,b). In addition, further research is needed to elucidate the underlying causes of such behaviors. To further explore gender differences among Pakistani university students in the current study, some differences between genders have also been reported.

### 2.3 Motivations behind bullying perpetration

The previous studies explored the motivations behind bullying perpetration in different groups, including school children (Fluck, 2017; Jaber et al., 2023; Tam and Taki, 2007; Sarıçam and Çetinkaya, 2018; Schreiner, 2019; Van Cleave and Davis, 2006; van Dijk et al., 2017), young adults (Abbasi et al., 2018; Johnston et al., 2014; Tanrikulu and Erdur-Baker, 2021; Xiu et al., 2021), and working professionals (Pheko, 2018; Siddiqui and Schultze-Krumbholz, unpublished study). Examining these different age groups helps to identify potential motives behind bullying perpetration. However, due to the lack of empirical research focusing specifically on the target population of university students, this study also included an age group that has not been extensively researched in terms of perceived motivations for bullying perpetration. Additionally, previous studies on young adults have primarily focused on bullying as a means to gain power, achieve social status (Abbasi et al., 2018; Xiu et al., 2021), or seek revenge (Johnston et al., 2014; Tanrikulu and Erdur-Baker, 2021). However, other motivational factors, such as using bullying to vent frustration, seek sensation or entertainment, or imitate the behavior of others, have not been thoroughly investigated. Therefore, the current study examines a broader range of perceived motivations within the target population.

Jaber et al. (2023) proposed a model that elucidates the underlying reasons for engaging in bullying perpetration. The researchers hypothesized that self-concept (e.g., self-esteem, self-confidence, feelings of inadequacy, and ego strength) and psychological adjustment (e.g., sensation seeking) influence attitudes and beliefs (e.g., moral reasoning about aggression) and perceived control (e.g., anger control and locus of control). These factors, in turn, influence intentions (both reactive and proactive) to engage in bullying perpetration. International research (Fluck, 2017; Pheko, 2018; Xiu et al., 2021) as well as studies conducted in Pakistan (Abbasi et al., 2018; Siddiqui and Schultze-Krumbholz, unpublished study) have elucidated the self-concept of the bullying mechanism as a quest for power or social status, which is identified as the primary motivation for reinforcing the self-ego. These studies have confirmed that individuals engage in aggressive behavior to demonstrate dominance and strength, an intrinsic drive for superiority as described by Sigmund Freud (Gay, 1999). The notion of gaining popularity and demonstrating strength is further elaborated by Garandeau and Cillessen (2006), who found that individuals who engage in bullying perpetration often enjoy elevated social status. This popularity attracts bystanders who may imitate the bullies in order to gain similar social influence, which is reinforced by both the bully’s celebrity and the fear of becoming a subsequent target. Another motivation for bullying, elucidated by Siddiqui and Schultze-Krumbholz (unpublished study), is rooted in social learning theory, which posits that individuals imitate behaviors in order to gain prestige comparable to that of the bullies.

In terms of psychological adjustment, researchers have identified sensation seeking, sadism, and the pursuit of entertainment as significant factors that lead bullying students to engage in violent and aggressive behaviors (Fluck, 2017; Schreiner, 2019; Siddiqui and Schultze-Krumbholz, unpublished study). Sensation seeking is a personality trait characterized by a desire for novel, exciting, and intense experiences. Individuals high in sensation seeking tend to pursue activities or situations that provide substantial stimulation and excitement (Zuckerman, 2014). By engaging in violent behavior, bullies achieve heightened levels of excitement and stimulation, which in turn satisfies their psychological needs and derives pleasure (Jaber et al., 2023; Siddiqui and Schultze-Krumbholz, unpublished study).

The model proposed by Jaber et al. (2023) elucidates that perceived control over emotions can lead to feelings of frustration and a desire for revenge, culminating in violent behavior toward others. Dollard et al. (1939) attributed hostility to frustration and dissatisfaction, while Buss (1961) posited that harmful actions toward others serve as a mechanism for relieving frustration. A widely accepted premise for bullying behavior is that it is triggered by external stressors (Siddiqui and Schultze-Krumbholz, unpublished study). Empirical evidence supports that bullying functions as a defense mechanism activated by external stressors to reduce anxiety (Tam and Taki, 2007). This framework suggests that frustration may reduce perceived control and provoke anger, resulting in aggressive behavior. Similarly, acts of revenge or retaliation are consistent with Jaber et al.'s (2023) concept of perceived control. Tanrikulu and Erdur-Baker (2021), in a cross-sectional study, identified revenge as a primary motive for bullying and inflicting harm on others. Sarıçam and Çetinkaya (2018) further elaborated that bullying also serves as a means to retaliate against provocateurs and exact revenge. When bullying is characterized as an act of revenge, it refers to the phenomenon in which victims of bullying often become perpetrators themselves (Van Cleave and Davis, 2006). Johnston et al. (2014) reported that after experiencing victimization, bully-victims derived a sense of improved self-esteem from bullying others. This finding suggests that one of the motivations for these individuals to engage in bullying is a desire for revenge.

Researchers have noted that bullies and bully-victims occupy different social positions within their classes, leading to the theorization that their bullying behaviors may be driven by different motivations (van Dijk et al., 2017). It has been suggested that bullies are primarily motivated by proactive reasons, such as gaining social status or asserting control, whereas bully-victims are driven by reactive motivations, such as anger or the need to fend off perceived social threats (Rodkin et al., 2015; Vlachou et al., 2011). In line with this argument, the current study seeks to elucidate the differences in perceived motivations between bullies, victims, bully-victims and non-responders (bystanders) that compel bullies and bully-victims to engage in bullying perpetration.

### 2.4 Role of peers and teachers in bullying intervention

Teachers are seen as key agents who can shape the institutional environment and use their skills to reduce bullying and victimization (Strohmeier et al., 2012). Similarly, peer-led programs have been developed in various regions where peers are trained to participate in events aimed at reducing bullying (Espelage and Hong, 2017). Many of these programs have demonstrated successful outcomes due to the involvement of peers in anti-bullying efforts (Menesini et al., 2018; Zambuto et al., 2020). Teacher-led interventions have also shown positive outcomes, although some programs have had limited success (van Verseveld et al., 2019). Previous research in Pakistan has highlighted that teachers are often not adequately trained to intervene in bullying incidents (Hakim and Shah, 2017; Shamsi et al., 2019; Siddiqui et al., 2023a). Similarly, peer-supported interventions are also largely absent in the Pakistani context (Siddiqui and Schultze-Krumbholz, 2023b). Moreover, most studies have focused on school teachers and younger students (Siddiqui and Schultze-Krumbholz, 2023b; Zambuto et al., 2020; Zhao and Chang, 2019). To fill this gap, the current study explores the role of teachers and peers in bullying intervention at the university level.

## 3 Research questions

This research addresses several key questions regarding bullying involvement among university students in Pakistan:

- Examines the prevalence of different bullying involvement groups, including bullies only, victims only, bully-victims, and non-responders.
- Identifies differences in moral disengagement beliefs among the various bullying groups.
- Investigates how perceptions of bullying motivations vary between different bullying groups.
- Explores the relationship between moral disengagement beliefs and motivations toward bullying.
- Examines the role of peers and teachers in fostering a safe educational environment at the university level, the degree of fear of victimization within the institution, the tendency to engage in perpetration, and students’ reactions to bullying incidents.

## 4 Methodology

This research study used a cross-sectional survey method and quantitative research design. The study aimed to determine the prevalence of bullying, moral disengagement beliefs, and perceptions of university students regarding the motivations behind the perpetration of bullying in educational institutions in Pakistan. Students were categorized into different bullying involvement groups based on their responses to the bullying and victimization items. In addition, demographic data (particularly gender differences) were used to examine differences in levels of moral disengagement and perceptions among different bullying groups. SPSS version 27 and AMOS 24 were used for analysis.

### 4.1 Sample and sampling procedure

Data was collected by sending Google Forms to department heads at various institutions across Pakistan, asking them to share the link with their students. As the forms were sent to department heads at several universities, who then forwarded them to students, the exact number of forms distributed is unknown and a response rate could not be calculated. At the end of the survey, 1,034 forms were returned and used for analysis. The sampling technique was both convenient and purposive, targeting only university students, as clearly stated in the instructions. The researchers adhered to basic ethical principles and the APA Code of Ethics. Students were informed of the purpose of the study, the voluntary nature of participation, and their right to withdraw at any time without consequence. Participants were required to acknowledge informed consent before proceeding to the questionnaire and providing their perceptions.

The participants of the study (male = 35%, female = 64%) were university students, including 628 undergraduate students, 212 graduate students, 131 M.Phil. research students, and 63 Ph.D. candidates, from 84 universities in different regions of Pakistan. The largest representation was from the Punjab region (*N* = 608), which is also the most populous province of the country. After Punjab, KPK had the second highest number of respondents (*N* = 163), followed by Sindh (*N* = 138) and Balochistan (*N* = 83). There were 17 respondents from Kashmir and 13 from Gilgit-Baltistan/FATA. Only 10 students responded from the Islamabad Capital Territory and 2 students provided data from other regions. For remaining demographics see Table 1.

**Table 1:** Demographics of the participants (age and gender).

Gender	Less than 18 years	18–25	26–33	34–41	42–49	50 and above	Total
Male	4	243	66	32	12	4	361
Female	5	546	64	42	6	2	665
Not disclosed	0	6	2	0	0	0	8
Total	9	795	132	74	18	6	1,034

### 4.2 Instrument

The two main tools used for research study are:

1. The Revised Olweus Bully/Victim Questionnaire (ROBVQ) (Olweus, 1996) is an instrument designed to assess the prevalence of bullying perpetration and victimization. It covers various forms of bullying, including verbal abuse, exclusion, physical aggression, spreading false rumors, stealing or damaging personal property, coercion or threats, and racial harassment. The questionnaire consists of 40 items measuring the extent of bullying/victimization, types of bullying (physical, verbal, indirect, racial, excluding sexual harassment for cultural reasons, example statement for victimization: *I was called mean names, was made fun of, or teased in a hurtful way;* example statement for perpetration: *I kept him or her out of things on purpose, excluded him or her from my group of friends or completely ignored him or her*), involvement in bullying perpetration, locations where bullying occurs, attitudes toward bullying, and awareness of and reactions to bullying incidents by teachers, peers, and parents. The Likert scale used in the questionnaire varies across different items. The sub-variables used in the current study from the ROBVQ are as follows:
  - Victimization: Measured using 10 items on a 5-point Likert scale (1 = “It has not happened to me in the past couple of months” to 5 = “It happened several times a week”).
  - Perpetration: Assessed with 10 items on a 5-point Likert scale (1 = “I have not bullied another student(s) at school in the past couple of months” to 5 = “I bullied several times a week”).
  - Bullying Grouping Dynamics: A single-item variable measured on a 6-point Likert scale (1 = “It has not happened to me in the past couple of months” to 6 = “By several different students or groups of students”).
  - Teacher Intervention: A single-item variable measured on a 5-point Likert scale (1 = “Almost never” to 5 = “Almost always”).
  - Bystander Intervention: A single-item variable measured on a 5-point Likert scale (1 = “Almost never” to 5 = “Almost always”).
  - Tendency to Involve in Bullying: A single-item variable measured on a 5-point Likert scale (1 = “Yes” to 5 = “Definitely No”).
  - Reactions Toward Bullying: A single-item variable measured on a 6-point Likert scale (1 = “I have never noticed that students my age have been bullied” to 5 = “I try to help the bullied student in one way or another”).
  - Fear of Victimization in the Institution: A single-item variable measured on a 5-point Likert scale (1 = “Never” to 5 = “Very often”).

- For single-item variables, the specific statements can be found in Table 2.

1. The Sohanjana Motivations behind Bullying Questionnaire (SMBBQ), developed and validated by Siddiqui and Schultze-Krumbholz (unpublished study), is designed to assess moral disengagement beliefs and perceived motivations for bullying perpetration. The scale consists of 32 items measured on a 5-point Likert scale (1 = strongly disagree, 5 = strongly agree) and assesses six variables.
  - Moral Disengagement (8 items, example statement: *Bully believes that talking bad-mouthed behind a person’s back is okay because he/she does not notice anything after all*)
  - Power Imbalance (8 items, example statement: *Bully wants to control others through bullying acts*)
  - Frustration causes Aggression (8 items, example statement: *Bully loses temper easily and gets irritated on little things*)
  - Bullying as Revenge (2 items, example statement: *A victim feels dishonored when he/she cannot get revenge*).
  - Bullying as Social Learning (2 items, example statement: *Bullying behavior is learned from observing and imitating role models, especially people with whom the learner has close and frequent contact with and who accepts and reinforces this behavior*)
  - Bullying as Sadism, Sensation Seeking and Entertainment (4 items, example statement: *Bullying results in the feeling of being turned up and stimulated*).

**Table 2:** Descriptive statistics.

Statement	Options	Frequency	Percentage
By how many students have you usually been bullied?	It has not happened to me in the past couple of months	810	78.3
	Mainly by 1 student	76	7.4
	By a group of 2–3 students	91	8.8
	By a group of 4–9 students	24	2.3
	By a group of more than 9 students	13	1.3
	By several different students or groups of students	20	1.9
How often do the teachers or other adults at your institution try to put a stop to it when a student is being bullied?	Almost never	257	24.9
	Once in a while	119	11.5
	Sometimes	126	12.2
	Often	191	18.5
	Almost always	341	33.0
How often do other students try to put a stop to it when a student is being bullied at your institution?	Almost never	228	22.1
	Once in a while	146	14.1
	Sometimes	195	18.9
	Often	245	23.7
	Almost always	220	21.3
Do you think you could join in bullying a student whom you do not like?	Yes	48	4.6
	Yes, May be	83	8.0
	I do not know	66	6.4
	No	380	36.8
	Definitely No	457	44.2
How do you usually react if you see or understand that a student your age is being bullied by other students?	I have never noticed that students my age have been bullied	322	31.1
	I take part in the bullying	25	2.4
	I do not do anything, but I think the bullying is OK	18	1.7
	I just watch what goes on	32	3.1
	I do not do anything, but I think I ought to help the bullied student	240	23.2
	I try to help the bullied student in one way or another	397	38.4
How often are you afraid of being bullied by other students in your school?	Never	447	43.2
	Seldom	187	18.1
	Sometimes	167	16.2
	Often	118	11.4
	Very Often	115	11.1

In its previous evaluation, the instrument was used to assess teachers’ perceptions of various motivations behind bullying and moral disengagement (Siddiqui and Schultze-Krumbholz, unpublished study). In this study, however, it is used to measure the perceptions of university students.

## 5 Data analysis and results of the study

### 5.1 Factor analysis

Before proceeding with data analysis and hypothesis testing, both instruments underwent factor analysis to ensure that they measured their intended constructs and had acceptable validity and reliability. Preliminary analyses indicated that the sample sizes for both instruments were adequate, as evidenced by KMO values greater than 0.7 (0.954 for the ROBVQ and 0.977 for the SMBBQ) (Kaiser and Rice, 1974; Leech et al., 2005). In addition, exploratory factor analysis was warranted because Bartlett’s test of sphericity was significant for both the ROBVQ (*χ*^2^(190) = 12,960.896, *p* < 0.001) and the SMBBQ (*χ*^2^(496) = 26,653.880, *p* < 0.001) (Hair et al., 2006; Bartlett, 1950). The results of the factor analysis and the factor loadings are shown in Table 3.

**Table 3:** Factor loadings.

Instrument 1: Revised Olweus Bully/Victim Questionnaire (ROBVQ)^a^				
Variable	Items	Factor loadings	Reliability Cronbach alpha	Validity AVE
Victimization	V1	**0.633**	0.889	58%
	V2	**0.747**		
	V3	**0.585**		
	V4	**0.558**		
	V5	**0.729**		
	V6	**0.627**		
	V7	**0.671**		
	V8	**0.630**		
	V9	**0.623**		
	V10	**0.595**		
Perpetration	P1	**0.663**	0.937	
	P2	**0.741**		
	P3	**0.715**		
	P4	**0.760**		
	P5	**0.797**		
	P6	**0.797**		
	P7	**0.802**		
	P8	**0.777**		
	P9	**0.776**		
	P10	**0.636**		

Instrument 2: Sohanjana Motivations behind Bullying Questionnaire (SMBBQ)^b^				
Variable	Items	Factor loadings (Standardized Regression Weights)	Reliability Cronbach alpha	Validity AVE
Moral disengagement beliefs	MD1	0.735	0.893	72%
	MD2	0.744		
	MD3	0.732		
	MD4	0.688		
	MD5	0.739		
	MD6	0.699		
	MD7	0.695		
	MD8	0.712		
Power imbalance	PI1	0.741	0.938	
	PI2	0.760		
	PI3	0.826		
	PI4	0.854		
	PI5	0.830		
	PI6	0.755		
	PI7	0.860		
	PI8	0.854		
Frustration causes aggression	FA1	0.777	0.943	
	FA2	0.832		
	FA3	0.840		
	FA4	0.859		
	FA5	0.840		
	FA6	0.850		
	FA7	0.841		
	FA8	0.748		
Bullying is revenge	BR1	0.823	0.809	
	BR2	0.826		
Social learning theory	SLT1	0.796	0.789	
	SLT2	0.818		
Sensation Seeking, Sadism and Entertainment	SSE1	0.794	0.891	
	SSE2	0.817		
	SSE3	0.869		
	SSE4	0.799		

a Extraction Method: Principal Component Analysis.

Rotation Method: Varimax with Kaiser Normalization.

b Standardized Regression Weights measured using AMOS. Values in bold indicate significant differences.

Given that the SMBBQ is a relatively new instrument that has only been used once to measure teacher perceptions (Siddiqui and Schultze-Krumbholz, unpublished study), it is important to ensure model fit criteria for this new instrument. Therefore, a model fit table was created and the values are presented in Table 4.

**Table 4:** Model fitness for SMBBQ.

Model fit	Recommended value	Final value	Reference
RMSEA	<0.07	0.058	Steiger (2007)
IFI	>0.90	0.942	Bollen (1989)
CFI	>0.90	0.942	Bagozzi and Yi (1988)
PCFI	>0.50 Higher the better	0.853	Schermelleh-Engel et al. (2003)
PNFI	>0.50 Higher the better	0.838	

### 5.2 Prevalence of bullying and victimization

One of the main objectives of the study was to assess the status of bullying and victimization in different regional higher education institutions in Pakistan. To achieve this, the Revised Olweus Bully/Victim Questionnaire (ROBVQ) scale for assessing victimization and perpetration was used to categorize students into four groups. Students who were targets of one type of bullying at least twice or two different types of bullying at least once in the past 6 months were classified as victim only. Similarly, students who bullied others in the same way at least twice or in two different ways at least once were classified as bully only. Students who were both bullied and involved in bullying others at least twice for one type of bullying or at least once for two types of bullying within the past 6 months were classified as bully-victim. Finally, a large number of students who were neither bullies nor victims were classified as non-involved. The distribution of students is presented in Table 5. The results show that 24% of the students were victimized and 14% were perpetrators of bullying in addition to being victims themselves. A large number of students (*N* = 611, 59.1%) were neither involved in perpetration nor victimization.

**Table 5:** Bullying classification.

Categorization	Gender	Frequency	Percent
Non-involved	Male = 191 Female = 416 Gender not indicated = 4	611	59.1
Victim-only	Male = 90 Female = 157 Gender not indicated = 3	250	24.2
Bully-only	Male = 12 Female = 12 Gender not indicated = 0	24	2.3
Bully-victim	Male = 68 Female = 80 Gender not indicated = 1	149	14.4
Total		1,034	100.0

### 5.3 Students perceptions about motivations behind bullying perpetration

One of the research questions was to explore university students’ perceived motivations for engaging in bullying perpetration. The SMBBQ, consisting of 24 items for perceived motivations, was used to address this question. After factor analysis, the items with high loadings from each factor were examined, fitted together and then presented in five sub-variables (perceived motivations): 8 items for power imbalance, 8 items for frustration causes aggression, 2 items for bullying as revenge, 2 items for bullying as social learning and 4 items for bullying as sensation seeking, sadism and entertainment. Responses were recorded on a 5-point Likert scale.

Descriptive statistics were used to answer this question. Mean scores indicated that most students believed that frustration was the primary reason for engaging in aggressive acts (*M* = 3.3207; SD = 1.168). The second most common response was the desire to demonstrate power and dominance over others (*M* = 3.3062; SD = 1.204). A significant number of students also perceived bullying as an act driven by sadism, sensation seeking, and entertainment, with students humiliating and mocking others for amusement (*M* = 3.2507; SD = 1.142) (see Table 6). However, how these beliefs differed between bullying groups will be explored in the later part of this study.

**Table 6:** Descriptive statistics for motivations behind bullying perpetration.

Variable	*N*	Minimum	Maximum	Mean	Std. Deviation
Frustration causes aggression	1,034	1.00	5.00	3.3207	1.16751
Power imbalance	1,034	1.00	5.00	3.3062	1.20383
Sensation seeking, sadism and entertainment	1,034	1.00	5.00	3.2507	1.14152
Social learning theory	1,034	1.00	5.00	3.1900	1.19490
Bullying is revenge	1,034	1.00	5.00	3.1833	1.19454

### 5.4 Impact of moral disengagement on different perceived motivations behind bullying

Another research question in this study was whether moral disengagement beliefs are correlated with different perceived motivations for bullying perpetration. Pearson’s correlations were computed using SPSS. Table 7 shows that Moral Disengagement is positively correlated with all the motivations listed, with the strongest correlation observed with Power Imbalance (PI) (*r* = 0.596, *p* < 0.01) indicating that students with higher levels of moral disengagement are more likely to perceive power imbalance as an important motive for bullying perpetration. The second strongest correlation is with Frustration causes Aggression (FA) (*r* = 0.534, *p* < 0.01), suggesting that students with higher levels of moral disengagement are more likely to see frustration as a trigger for aggression and bullying perpetration. Following this, Sensation Seeking, Sadism and Entertainment (SSE) (*r* = 0.513, *p* < 0.01) shows a third strongest correlation, suggesting that higher moral disengagement may be associated with sadistic enjoyment or entertainment through bullying.

**Table 7:** Pearson’s correlation (impact of moral disengagement on different motivations).

Variables	MD	PI	FA	BR	SLT	SSE
Moral disengagement (MD)	1	0.596^**^	0.534^**^	0.446^**^	0.486^**^	0.513^**^

Power Imbalance (PI), Frustration causes Aggression (FA), Bullying is Revenge (BR), Social Learning Theory (SLT), Sensation Seeking, Sadism and Entertainment (SSE).

**Denotes results that are significant at the 0.01 level.

### 5.5 Association of the status of bullying and victimization with moral disengagement beliefs and perceived motivations behind bullying

The authors of the current study also wanted to see if there were differences in moral disengagement beliefs between students who had been victimized and those who had bullied others, those who had been involved in both bullying and victimization, and those who had not been involved. In addition, we wanted to understand whether these groups had similar perceptions of the motivations behind bullying. To explore these questions, a one-way ANOVA was conducted to identify any differences. The results indicated that there were significant differences in students’ beliefs about power imbalance and frustration as motives for aggression in bullying. However, no significant differences were found among the other variables (see Table 8).

**Table 8:** ANOVA (dependent variable: moral disengagement beliefs and motivations behind bullying perpetration).

		Sum of squares	Df	Mean square	*F*	Sig.
Moral disengagement beliefs	Between groups	4.829	3	1.610	1.327	0.264
	Within groups	1,249.256	1,030	1.213		
	Total	1,254.085	1,033			
Power imbalance	Between groups	11.733	3	3.911	2.712	**0.044**
	Within groups	1,485.297	1,030	1.442		
	Total	1,497.029	1,033			
Frustration causes aggression	Between groups	13.380	3	4.460	3.294	**0.020**
	Within groups	1,394.683	1,030	1.354		
	Total	1,408.063	1,033			
Bullying is revenge	Between groups	5.638	3	1.879	1.318	0.267
	Within groups	1,468.383	1,030	1.426		
	Total	1,474.021	1,033			
Social learning theory	Between groups	3.561	3	1.187	0.831	0.477
	Within groups	1,471.346	1,030	1.428		
	Total	1,474.907	1,033			
Sensation seeking, sadism and entertainment	Between groups	9.870	3	3.290	2.536	0.055
	Within groups	1,336.192	1,030	1.297		
	Total	1,346.062	1,033			

Independent Variable: Categorization of Students (Victim only, Bully only, Bully-Victim, Non-responsive).

Values in bold indicate significant differences.

The *post hoc* analysis, conducted using the Games-Howell method due to the significance of the Levene’s test (*p* < 0.05), revealed significant differences but with small effect size between students who were only victimized (victims only) and those who were both victimized and involved in bullying others (bully-victims) (see Table 9). Specifically, students who were only victimized (*M* = 3.41, SD = 1.16) had a stronger belief that the primary motivation for bullying was a power imbalance compared to students who were both victimized and involved in bullying others (*M* = 3.09, SD = 0.977), but the effect size is small with a Cohen’s *d* value of 0.2. A similar pattern was observed regarding frustration as a motive for aggression with a Cohen’s *d* value of 0.1, with only victims (*M* = 3.45, SD = 1.13) having a stronger belief than bully-victims (*M* = 3.10, SD = 1.02). No significant differences were found among the other variables. Another interesting finding from Table 9 was that there were larger mean differences in beliefs between bully only, victim only, and bully-victim groups. The mean scores indicated that students involved in bullying only (bully only) had lower beliefs in motivations such as frustration causes aggression (*M* = 3.02, SD = 1.07) and power imbalance (*M* = 3.01, SD = 1.14) compared to the other two groups. Mean scores indicated that entertainment, sensation seeking, and sadism were the primary motivations for the bully only group (*M* = 3.30, SD = 0.91). However, these differences were not statistically significant, likely due to the large difference in sample size between the bully only group and the other groups.

**Table 9:** Post-hoc analysis (multiple correlations using games Howell method).

Dependent variable	Category of student (I)	Category of student (J)	Mean difference (I-J)	Std. error	Sig.	95% confidence interval	
						Lower bound	Upper bound
Power imbalance	Victim only	Non-involved	0.07612	0.08959	0.831	−0.1548	0.3070
		Bully-only	0.40029	0.24426	0.374	−0.2669	1.0675
		Bully-victim	0.31238*	**0.10868**	**0.022**	0.0319	0.5929
Frustration causes aggression	Victim only	Non-involved	0.11453	0.08646	0.548	−0.1084	0.3374
		Bully-only	0.43237	0.22919	0.256	−0.1932	1.0579
		Bully-victim	0.34397*	**0.11008**	**0.010**	0.0598	0.6282

*The mean difference is significant at the 0.05 level.

Values in bold indicate significant differences.

### 5.6 The role of peers and teachers to create a safe environment at educational institutions

The final aim of the study was to examine students’ responses to bullying and perpetration within their institutions. To this end, several statements from the ROBVQ were adapted to the context of higher education institutions (see Table 2). The statements and Likert scale used varied across items, with details provided in Table 2 and section 4.2.

Bullying was most often perpetrated by a single student or a small group of 2–3 students, rather than by large groups. About 25% of students reported that teachers and administrators at their institutions did not take action to control bullying. In contrast, 33% of students reported that teachers and other adults consistently took responsibility and tried to control bullying perpetration. In addition, 22% of students reported that bystanders usually did not intervene during bullying incidents.

In addition, 12% of students expressed a willingness to join a bullying group if the victim was unpopular. A smaller percentage (about 7%) reported either participating in bullying or enjoying watching it, highlighting the role of the passive bystander. In addition, 22.5% of students confirmed their fear of being victimized at their institution, indirectly indicating the prevalence of frequent bullying episodes. Notably, the fear of being victimized at university was more prevalent among female students (*M* = 2.40, SD = 1.411) compared to their male counterparts (*M* = 2.10, SD = 1.40) (see Table 10). Although no significant differences were found in terms of victimization between male (*M* = 3.41, SD = 5.26) and female students (*M* = 2.90, SD = 5.75), the level of perpetration was higher for male students (*M* = 2.32, SD = 5.21) compared to female students (*M* = 1.62, SD = 5.24) (see Table 10).

**Table 10:** Independent sample t-test.

Factor: gender						
Factor	*F*-value	*T*	Df	Sig (2-tailed)	Mean difference	Standard error difference
Victimization	0.641	1.481	1,024	0.139	0.54056	0.36492
Perpetration	7.228	2.07	1,024	0.038	0.70906	0.34209
Fear of attending institute	5.54	−3.62	1,024	0.000	−0.33	0.091

## 6 Discussion

School bullying, a pervasive international problem, has received considerable attention from the global research community. Scholars worldwide have focused on various aspects of bullying, including interventions, anti-bullying policies, measurement of school bullying, and its association with related variables (Wong et al., 2013). In line with this, the current research aims to identify factors that contribute to bullying and propose effective interventions based on an understanding of the core causes of antisocial behavior. This study examines university students’ perceptions of the motivations behind bullying perpetration and the correlation of moral disengagement beliefs on various perceived motivations. It also examines the prevalence of bully roles among university students in Pakistan, as well as differences in their moral disengagement beliefs and perceptions of the motivations behind bullying perpetration. This study aimed to identify differences in the perceptions of students across four categories: bullies only, victims only, bully-victims, and non-involved. Johnston et al.’s (2014) study suggested that bully-victims’ motivations for bullying were similar to those of bully only individuals. In this line, the current study did not identify any significant differences in motivations between bullies and bully-victims. However, a notable difference was found between students categorized as victims only and those categorized as bully-victims, which will be discussed in the following section.

The study seeks to determine students’ perceptions of the motivations behind bullying. The results contrast with those of Siddiqui and Schultze-Krumbholz (unpublished study), who reported that teachers perceived the primary motivation for bullying to be the demonstration of power or strength, followed by frustration causes aggression. Conversely, students believe that those who bully are primarily motivated by frustration and view bullying as a way to vent their frustration on others. When the data were analyzed descriptively for all students combined (including bullies only, victims only, bully-victims, and non-responders), it was found that the majority believed that frustration was the primary motivation for bullying. Further analysis to see the differences in the beliefs of the groups revealed that victims, more than bullies and bully-victims, strongly believed that frustration was the main reason for bullying. This suggests that victims, despite being targets, may perceive that those who bully are dealing with negative circumstances, which drives their bullying behavior. This suggests that victims perceive bullies as frustrated individuals, which may foster a sense of sympathy and deter them from seeking revenge or engaging in bullying themselves. In contrast, bully-victims, who hold a weaker belief in frustration as a motivation, do not view bullies as merely frustrated individuals and are more inclined to replicate bullying behaviors as a form of retaliation for their own victimization. However, it is important to note that the sample size for the “bullies only” students was much smaller than the other two groups. As a result, despite the differences observed, statistical significance was not reached for the bully only group. Researchers have identified several factors that contribute to increased frustration within higher education institutions, including financial burdens, academic pressures, and parental academic expectations (Gulzar et al., 2012). In addition, students have been reported to be upset by rising inflation, unemployment, and uncertainty about the future (Khalid et al., 2021).

The collective data analysis revealed that overall university students also believe that the second most common reason for bullying in Pakistani educational institutions is a display of power, with bullies feeling superior, essentially reflecting a power imbalance. Some differences were observed when further analyzing the differences in perceptions of the different bullying groups. Specifically, it was found that students who were exclusively victimized had a stronger belief that the primary motivation for bullying was a power imbalance, in contrast to students who were both victimized and involved in bullying others and pure bullies. This pattern suggests that victimized students have resigned themselves to the belief that they are powerless against the bully due to the perceived power imbalance. Perpetrators of bullying typically externalize blame, attributing responsibility to others, whereas victims tend to internalize blame, often perceiving themselves as the cause of their victimization (Morrison, 2006). This internalization can lead victims to develop a belief in their own powerlessness, accept their victimization as a consequence of their perceived mistakes, and resign themselves to their fate. Conversely, bully-victims, who reject the notion of power imbalance or the superiority of bullies, retaliate by bullying others to demonstrate their own strength. The phenomenon of bullying as a means of demonstrating power and superiority in Pakistani society can be traced back to historical and cultural practices (Khan and Wazir, 2011; Siddiqui and Schultze-Krumbholz, unpublished study). Social class distinctions, established historically by figures such as maharajas and rajas (kings and great rulers), and in modern times by landlords, military personnel, bureaucrats, high-ranking officials, and even general male hegemony, have been reinforced through both acceptable and unacceptable behaviors, ensuring unquestioned compliance (Khan and Wazir, 2011; George et al., 2020; Hinduja et al., 2023). Caste, religion, and race are also significant factors contributing to differences and bullying behavior (Javed et al., 2023; Qamar et al., 2023). Shah et al. (2022) conducted a qualitative study showing that violence is often driven by caste or ethnic differences, with dominant and powerful castes (e.g., Chaudaries in Punjab) perpetrating violence against those perceived to belong to lower castes or ethnicities. In Pakistan, success is often equated with power, which may explain why some students engage in bullying to assert dominance and gain an advantage. The powerful and disciplined military and bureaucratic institutions that have historically looked down upon the political leadership of the newly formed state in contempt have also contributed to this power imbalance (Khan and Wazir, 2011).

The third most common reason students reported for bullying others was sensation seeking, entertainment, or sadism. It was also found that the bully only group was less likely than the other two groups (victims only and bully-victims) to believe that their bullying behavior was driven by power imbalance and frustration. The results suggest that most bullies believe that the driving forces behind their behavior are sensation seeking, entertainment, and a sadistic approach. However, due to the small sample size of the “bullies only” group, these differences did not reach statistical significance. These findings are consistent with the views of university teachers in Pakistan who believe that university students often bully others for thrills and entertainment (Siddiqui and Schultze-Krumbholz, unpublished study). This motivation has also been recognized by other international researchers (Fluck, 2017; Jaber et al., 2023; Schreiner, 2019). However, the current study shows that these beliefs vary among students depending on their specific bullying roles. One possible explanation for this behavior is that students have the privilege of attending university; to make their academic journey more exciting and thrilling, they inject an element of hostility into their interactions. Abdullah (2023) elaborates on this behavior by linking it to peer pressure. According to Abdullah (2023), university students experience significant peer pressure, which leads to sensation-seeking behaviors that are often expressed through bullying. Another possible reason for these behaviors is the lack of supervision and accountability for students’ actions at universities, which allows them to engage in such behaviors with minimal consequences. In addition, university curricula focus more on skill development than on the moral or ethical development of students. Therefore, it is crucial to address moral disengagement beliefs in interventions to guide youth and emphasize that such behaviors are unacceptable and have long-term consequences for victims.

The results of the study also contrast with the study conducted by Pšunder and Kozmus (2020), which posited that bullying is an act of revenge. In contrast, the current study shows that many students do not perceive bullying as an act of retaliation for previous victimization. This discrepancy may be attributed to Muslim students’ belief in karma, which leads them to refrain from seeking revenge because they believe that Allah (God) will provide the best retribution. In addition, the religious teachings, which advocate forgiveness over revenge, may influence this perspective.

In the current study, the number of victims was slightly less than twice the number of bullies, suggesting that victims are less likely to seek revenge or retaliation. This may be due to their religious beliefs and the concept of karma, which discourage them from retaliating or becoming perpetrators.

Another goal of the study was to determine the relationship of moral disengagement beliefs on various perceived motivations of bullying. Numerous studies have found a relationship between moral disengagement and bullying perpetration (Teng et al., 2020). The findings of the present study are consistent with previous research indicating that children and young adults with higher levels of moral disengagement are primarily motivated to engage in bullying perpetrations in order to assert dominance and gain pleasure (Fluck, 2017; Siddiqui and Schultze-Krumbholz, unpublished study). In contrast to the findings of Siddiqui and Schultze-Krumbholz (unpublished study), this study also suggests that moral disengagement influences frustration as a cause of bullying. This implies that students with weak moral convictions are more likely to rationalize antisocial behavior as acceptable when experiencing frustration.

The results of the current study indicate that university students are most often categorized as non-involved, followed by victims only, bully-victims, and then bullies only. These findings contrast with the study by Khawar and Malik (2016), which found that bully-victims were the most common group among school students. One possible explanation for this discrepancy is that school children, who are more energetic and confident, are more likely to seek revenge, a tendency that decreases as students mature as explained by Allemand and Olaru (2021). Consequently, university students appear to be more susceptible to victimization and less likely to engage in bullying as a response to their own victimization. It is also noteworthy that approximately 14% of students were classified as bully-victims. Among all individuals involved in bullying perpetration, bully-victims have the poorest psychosocial outcomes (Jaber et al., 2023). They have been found to be at higher risk for depression and suicidal ideation (Pranjic and Bajraktarevic, 2010), as well as non-suicidal self-injury (Espositon et al., 2019). Therefore, it is imperative that this group of students receive special attention in intervention programs.

Previous research has shown that adolescents who are involved in bullying generally have a higher tendency toward moral disengagement beliefs compared to non-involved adolescents (Gini et al., 2014; Runions et al., 2019; Thornberg and Jungert, 2014). These studies reported that moral disengagement was prevalent among both pure bullies and bully-victims compared to students who either did not report involvement in bullying or only reported being victims (Runions et al., 2019). However, in the current study, no significant differences were found among the four categories of students reported (victims only, bullies only, bully-victims, or non-responders). These findings are unexpected given that the majority of the population recognizes the moral wrongness of bullying based on religious beliefs and cultural teachings. Possible reasons for not finding differences in moral disengagement beliefs may be related to the items used in the newly developed questionnaire, which reflect generally morally acceptable practices in Pakistani society, even though they may be considered morally wrong in other cultures. For example, one item categorized passive bystander behavior as morally disengaged, yet this behavior is often justified by the belief that it is not the bystander’s responsibility to intervene and protect the victim from bullying. Adults often instruct individuals to avoid getting involved in conflicts or problems under the guise of helping others. While not intervening in such situations is considered morally wrong internationally, it is often accepted in Pakistani society, where bystanders typically avoid involvement to protect themselves from potential trouble. This perception is widespread in society, which may explain why bystanders do not intervene, as noted by Siddiqui and Schultze-Krumbholz (2023a,b). Future studies should compare moral disengagement items with previously tested, validated, and reliable questionnaires to identify and improvise these items to truly measure moral disengagement beliefs and differences among different groups. Nevertheless, bullying persists, suggesting that individuals may dissociate from their moral values in order to engage in such behavior. Further research is recommended to clarify these discrepancies.

Descriptive statistics from the ROBVQ shed light on the prevalence of bullying in higher education institutions, the role of teachers and peers in intervention, and the extent to which bullying induces anxiety in students about attending university. Although this was not the primary objective of the research, a brief discussion is warranted to underscore the complexity of this issue in a country where bullying research is still in its infancy. The results showed that a significant proportion of students (24%) reported experiencing victimization at their university. These findings show both congruence and divergence with previous studies conducted in Pakistan. For example, Musharraf and Anis-ul-Haque (2018) reported that more than 60% of university students engaged in bullying perpetration, while Ayub et al. (2022) found that approximately 70% of postgraduate trainees encountered bullying at their institution. Similarly, Saleem et al. (2021) reported that approximately 90% of university students experienced bullying. The comparatively lower prevalence in the current study may be due to the strict criteria used (victimization occurring at least twice in 6 months). In addition, Bjereld (2018) noted that victims often avoid being labeled, may be in denial, or may perceive bullying as an everyday problem. Some victims may be embarrassed to disclose their experiences and therefore refrain from reporting victimization, while others may be unsure whether the bully’s behavior constitutes true bullying or mere pranks. Despite assurances of anonymity in the current study, it is plausible that students were reluctant to reveal their true suffering, resulting in a lower incidence of reported victimization. A similar conclusion can be drawn from the approximately 22% of students who regularly fear attending their university because of the risk of victimization. In addition, there is a notable difference in the fear of attending university, which is more prevalent among female students. These numbers are alarming, especially in a country where nearly half of the population is illiterate (Rehman et al., 2015). If a quarter of the student population feels unsafe attending educational institutions, the literacy rate is likely to decline further. It is noteworthy that while both male and female students are equally victimized, there is a significant difference in perpetration, with male students more likely to be involved in bullying others. A similar finding has been reported by other researchers in Pakistan, showing that male participants were significantly more involved in bullying others compared to females (Abid et al., 2017; Khawar and Malik, 2016; Shahzadi et al., 2019). Comparable findings have been documented across cultures, indicating that perpetrator behaviors are more prevalent among male students than female students (Pšunder and Kozmus, 2020). This finding highlights the patriarchal nature of Pakistani society, where female victimization is more common and male participants are more likely to perpetrate on others, as highlighted by Hinduja et al. (2023). As a result, female students often feel more insecure about attending university for fear of victimization.

The study also highlights the role of faculty and bystanders in intervening in bullying incidents. Researchers found that nearly a quarter of students reported that bullying was prevalent, but university administrators and teachers did not act to control or intervene in such behaviors. Shamsi et al. (2019) reported that more than half of the teachers in Pakistan lacked adequate knowledge to address bullying issues, which are prevalent in educational institutions. This suggests that university teachers may also be untrained or lack the confidence to address such issues with students. In addition, university students tend to solve their problems independently (Siddiqui et al., 2023a), which may contribute to the lack of teacher involvement in addressing antisocial behavior in higher education institutions. The study also highlighted the role of passive bystanders, with nearly a quarter of students reporting that bystanders typically do not intervene in bullying incidents. In addition, a small number reported participating in bullying incidents by accompanying the perpetrator or enjoying such incidents. These findings are consistent with the study by Siddiqui and Schultze-Krumbholz (2023a), who found that students in Pakistani educational institutions are often reluctant to intervene. Siddiqui and Schultze-Krumbholz (2023a) attribute this behavior to several factors, including fear of victimization for reporting or intervening, lack of knowledge about how to respond, being instructed to avoid involvement, and moral disengagement beliefs. Further research is recommended to determine the reasons for the lack of intervention.

The results also suggest that the majority of students experienced bullying perpetrated by a small group of peers. This observation is consistent with the findings of Siddiqui and Schultze-Krumbholz (2023b), who reported that most students identified their bullies as a small group of 2–3 individuals.

## 7 Implications

Bullying is a complex phenomenon that has been addressed through a variety of strategies. Previous researchers (Gini et al., 2014; Runions et al., 2019; Siddiqui et al., 2023b) and the authors of the current study have advocated for the inclusion of moral disengagement interventions in anti-bullying intervention programs. While not explicitly designed to alter moral disengagement, the innovations in the programs aim to assess whether individual and collective moral disengagement influences bullying perpetration and mediates changes in such behaviors in schools. Identifying the fundamental underlying mechanisms that drive behavioral change away from bullying remains a central goal of bullying prevention and intervention efforts.

Researchers have also developed different taxonomies of the motivations behind violence and bullying. For example, instrumental violence is also considered a motivation when the perpetrator attacks the victim to achieve a goal that cannot be achieved through nonviolent means (Fluck, 2017). In such cases, bullies often blackmail their victims into giving them money or valuable items. Another motive cited by Fluck (2017) is an ideological belief, which refers to in-group or out-group phenomena that lead to violence against people that students perceive as out-group members. When it comes to bullying, the characteristics that increase the likelihood of becoming a victim appear to be psychological (e.g., low self-esteem, shyness, introversion) rather than variables such as ethnicity, social status, physical appearance, sexual orientation, religious affiliation, and other obvious characteristics that differentiate the individual from the majority. It is therefore reasonable to assume that ideological reasons play a rather minor role. However, little is known about whether these motives play a significant role, and it is recommended that further research be conducted on these motives to find out whether or not this motivation is also prevalent in the general Pakistani society.

The purpose of the study was not only to determine the overall prevalence, but also to determine the percentage of students involved in different bullying roles and the differences in their perceptions. This highlights that the motivations and beliefs of different bullying roles differ, suggesting that interventions should be tailored based on these perceptions. It also leads to the recommendation that intervention programs should first establish a baseline to determine the prevalence of bullying roles, and then be adjusted and tailored according to the specific beliefs of those roles.

## 8 Ethical statement

Researchers adhered to basic ethical principles and followed the APA Code of Ethics. Participants gave informed consent via an online forum, which is equivalent to written consent. After reading details about the purpose of the study, anonymity, voluntary participation, planned use of data, and the right to withdraw without consequence, participants gave their explicit consent by clicking the “I agree” button at the beginning of the survey. This ensured informed consent in accordance with ethical guidelines and federal law. The study, which involved human participants, did not require ethical review and approval according to local laws and institutional requirements. The research was conducted as a survey rather than an experimental design, with the goal of gathering participants’ perceptions and beliefs about the motivations behind bullying. As a result, the study had no adverse effects on the participants. The second author’s research team reviewed and confirmed that the study adhered to basic ethical principles and ensured that no potential harm was caused to participants. The review team found no potential conflicts of interest, harm to participants, or activities that deviated from ethical codes of conduct.

## 9 Limitations and direction for future research

This study focused exclusively on university students and is cross-sectional in nature. It is recommended that future research includes additional age groups, such as high school and college students, to further explore differences in perceived and real bullying motivations and their relationship to moral disengagement across educational levels. In addition, a longitudinal study should be designed to assess changes in bullying roles and perceptions of bullying motivations. Some of the results are purely descriptive, and more advanced statistical models, such as structural equation modeling, will be necessary in the future to explore the complex interrelationships in greater depth. It is important to note that this study captured students’ perceived reasons for bullying, including those who were not directly involved in bullying episodes, such as bystanders or uninvolved students. These perceptions may differ from the actual reasons for bullying. One contributing factor is the limited number of students classified as bullies or bully-victim, probably due to the self-report nature of the questionnaire. To address this, future studies could involve teachers or peers to help identify students in the role of bully or bully-victim. Gathering opinions about motives directly from these identified groups could provide a more accurate understanding of the true motivations behind bullying. Therefore, it is recommended that future research incorporates data triangulation to accurately identify bullies and capture their true motivations.

## Data Availability

The raw data supporting the conclusions of this article will be made available by the authors without undue reservation.

## Footnotes

^1^Siddiqui, S., and Schultze-Krumbholz, A. (unpublished study). Sohanjana Motivations behind Bullying Questionnaire (SMBBQ): First version of an instrument to measure teachers’ perceptions.
